# Identification of a Biomarker Panel for Diagnosis of Early Childhood Caries Using Salivary Metabolic Profile

**DOI:** 10.3390/metabo13030356

**Published:** 2023-02-27

**Authors:** Seonghye Kim, Yuri Song, Seyeon Kim, Siyeong Kim, Heesam Na, Sujin Lee, Jin Chung, Suhkmann Kim

**Affiliations:** 1 Department of Chemistry and Chemistry Institute for Functional Materials, Pusan National University, Busan 46241, Republic of Korea; 2 Department of Oral Microbiology, School of Dentistry, Pusan National University, Yangsan 50612, Republic of Korea; 3 Oral Genomics Research Center, Pusan National University, Yangsan 50612, Republic of Korea; 4 Department of Dental Hygiene, Jinju Health College, Jinju 52655, Republic of Korea; 5 Dental Research Institute, BK21 PLUS Project, School of Dentistry, Pusan National University, Yangsan 50612, Republic of Korea

**Keywords:** dental caries, saliva, metabolic profiling, nuclear magnetic resonance spectroscopy

## Abstract

Several studies have demonstrated that nuclear magnetic resonance (NMR) metabolic profiles can differentiate patients with caries from healthy individuals; however, these studies only identified individual metabolites. The present study aimed to identify a salivary metabolite biomarker panel for the diagnosis of early childhood caries (ECC). Saliva samples from children with and without caries were analyzed using NMR spectroscopy. Multivariate and univariate analyses were performed to identify the discriminating metabolites. Selected metabolites were further evaluated and used to detect ECC. The saliva samples of children with ECC were characterized based on the increased levels of formate, glycerophosphocholine, and lactate and reduced levels of alanine, glycine, isoleucine, lysine, proline, and tyrosine. The levels of these metabolites were significantly different from those in the control in the ECC subgroup according to caries severity and correlated with the number of decayed and filled teeth or surfaces. Subsequently, an optimal salivary metabolite biomarker panel comprising formate, lactate, proline, and glycine was developed. This panel exhibited a better diagnostic performance for ECC than a single metabolite. These results demonstrate that salivary metabolic signatures can reflect oral conditions associated with dental caries, thereby emphasizing the importance of distinct salivary metabolic profiles as potential biomarkers of ECC.

## 1. Introduction

Early childhood caries (ECC) is defined as the presence of one or more decayed, missing, or filled tooth surfaces in any primary tooth in a child under six years of age or younger [1]. According to the 2018 Korean Children’s Oral Health Survey, >60% of children aged five years have experienced dental caries, and the prevalence of ECC has shown an increasing trend since 2016 [2]. ECC negatively impacts a child’s quality of life by causing pain, infection, and abscesses, and can also affect lifelong oral health by increasing the risk of developing new carious lesions in the permanent dentition or periodontal disease [3,4]. 

Dental caries results from enamel demineralization by organic acids produced by biofilm-forming bacteria generated from the metabolism of dietary fermentable carbohydrates, primarily sugars. An increased frequency of sugar intake results in an oral environment with a lower pH, which increases the proportion and activity of acid-tolerating (pathogenic) bacteria [5]. Therefore, the consumption of sugars (i.e., sugars added to foods and beverages) is an important risk factor for ECC [6].

ECC can be prevented through diet counseling, parental education, good oral hygiene practices, and alleviated by using topical fluoride [7]. If proper management fails, young children may be treated even under deep sedation or general anesthesia [8]. There are several diagnostic techniques available including visual examination, visual-tactile examination, radiography, and laser fluorescence [9]. However, visual inspection can be influenced by the subjective judgment of the inspector, and radiographic examination has limitations in accurately and precisely determining the extension of carious lesions [10]. Moreover, laser fluorescence devices are only effective in detecting occlusal caries and may detect the presence of stains and plaque during measurements, thereby reducing the specificity for detecting enamel lesions [11]. Therefore, the development of early and accurate detection method for the diagnosis of ECC is required.

Saliva can reflect the oral physiological state of an individual because it directly contacts and surrounds the teeth, oral microbiota, and dental plaque. Various salivary factors, including pH, buffering capacity, proteins, and enzymes, have been widely studied as biomarkers of dental caries [12,13]. In addition, salivary metabolites, which are low molecular weight compounds (<1 kDa), are promising biomarkers because they can provide an organism with a phenotype in a specific biological state. Nuclear magnetic resonance (NMR) is the main analytical platform for metabolic profiling of biofluids, with several advantages such as high reproducibility, rapid and non-destructive measurement, and minimal sample requirement [14]. NMR-based metabolomic studies on saliva have comprehensively established quantifiable metabolite profiles [15,16,17], sample preparation methods [18,19], and influencing factors [20,21,22]. To date, several studies using NMR have identified differences in salivary metabolomes and discovered potential biomarkers for several diseases, including oral cancers [23,24], periodontitis [25,26,27], and neurodegenerative disorders such as Alzheimer’s and Parkinson’s diseases [28,29]. However, relatively few studies have been published on dental caries. Several studies have demonstrated that NMR metabolic profiles can differentiate patients with caries from healthy individuals [30,31]; however, these studies only identified individual important metabolites, and some of these showed conflicting results. Using multiple biomarkers rather than a single biomarker can improve performance and reduce misdiagnosis due to arbitrary changes in a single biomarker [32]. Therefore, this study aimed to identify discriminating metabolites for ECC and construct a highly robust multiple-biomarker panel. We also investigated the correlation between metabolite biomarkers and study participant characteristics associated with the development of caries.

## 2. Materials and Methods

### 2.1. Study Population

The child cohort comprised healthy children enrolled from community child centers in Yangsan, South Korea. Diagnosis of dental caries and dental examinations were performed based on the criteria of the International Caries Detection and Classification System (ICDAS). Experience of dental caries was measured by the number of decayed and filled teeth (dft) and surfaces (dfs) of all participants. To estimate caries activity, Snyder’s test was performed; the detailed method is provided in the Supplementary data. Information on the drink and snack consumption frequency, and tooth brushing frequency was obtained using a questionnaire. 

This study was approved by the Institutional Review Board of Pusan National University (IRB no. PNU 2018-049). Written informed consent was obtained from the parents or guardians of all study participants. All experiments were performed in accordance with the relevant guidelines and regulations.

### 2.2. Saliva Collection and Sample Preparation

Saliva samples were collected after informing the participants of the sampling protocol and prior to further oral examinations. All participants were asked to refrain from consuming food or drinks, brushing, or using a mouth wash for at least 1 h before sampling and were scheduled for saliva sampling between 9:00 AM and 11:00 AM. Stimulated whole saliva was collected using a hygiene collection system (Salivette; Sarstedt, Nümbrecht, Germany). A plain cotton roll was placed in the mouth of each participant for approximately 1 min to stimulate salivation. The cotton roll, after the absorption of a sufficient amount of saliva, was placed into a Salivette tube and immediately transported to the laboratory. The cotton roll was centrifuged at 1000× *g* and 4 °C for 20 min, and the supernatant was removed and stored at –80 °C.

### 2.3. NMR Measurements

Prior to NMR experiments, frozen saliva samples were thawed and centrifuged at 10,000 rpm for 1 min. Approximately 450 µL of the supernatant was mixed with 50 µL phosphate buffer (pH 7.4) in deuterated water (D_2_O, 99.9% D), containing 20 mM 3-(trimethylsilyl) propionic-2,2,3,3-d_4_ acid sodium salt (TSP-d_4_), and transferred into 5-mm NMR tubes for analysis. ^1^H NMR spectra were acquired using a 600 MHz NMR spectrometer (Agilent Technologies Inc., Santa Clara, CA, USA). The Carr–Purcell–Meiboom–Gill pulse sequence with presaturation was used. For each sample, the ^1^H NMR spectrum acquired with the following parameters: a spectral width of 9615.4 Hz, 128 transients, a relaxation delay of 3.0 s, a 90° pulse of 13.2 µs, a spin-echo delay of 500 µs, 66 number of loops, an acquisition time of 3.0 s, and a total acquisition time of 13 min and 9 s. All FIDs were zero-filled to 64k points and subjected to line broadening of 0.3 Hz. The acquired NMR spectra were phase and baseline corrected and subsequently referenced to the TSP peak at 0.0 ppm. 

### 2.4. Multivariate Statistical Analysis

The processed NMR spectra were binned from 0.75 to 8.5 ppm with a 0.01 ppm binning size, resulting in 635 bins for each spectrum. Regions of water signal suppression (4.2 to 5.6 ppm) were excluded. To compensate for the differences in sample concentration, each binned data point was normalized to the total area of the spectra.

Multivariate statistical analyses of ^1^H NMR spectral data were performed using the SIMCA-P software (version 12.0; Umetrics, Umeå, Sweden). All analyses applied Pareto scaling. First, principal component analysis (PCA) was applied to confirm the intrinsic variation and metabolic pattern of unsupervised samples. Orthogonal partial least squares-discriminant analysis (OPLS-DA) was performed to improve the discrimination between groups and identify distinguishing variables. The model quality was evaluated based on fitting (R^2^Y) ability, predictive (Q^2^Y) ability, and cross-validated analysis of variance (CV-ANOVA). The variable importance in projection (VIP) scores of the OPLS-DA model were used to identify the metabolites contributing to group classification. Spectral variables with high VIP scores and low jackknife standard errors (VIP_cvSE_) were considered significant.

### 2.5. Univariate Statistical Analysis and Receiver Operating Characteristic (ROC) Analysis of Selected Metabolites

Categorical (sex, drink and snack consumption frequency, and tooth brushing frequency) and continuous (age, height, and weight) characteristics were analyzed using the chi-square and t-tests using IBM SPSS Statistics 25 (SPSS, Inc., Chicago, IL, USA), respectively. 

Qualitative and quantitative analyses of salivary metabolites were conducted using Chenomx NMR suite 8.4 (Chenomx Inc., Edmonton, Canada). All metabolites were identified using the Human Metabolome Database (HMDB, www.hmdb.ca, accessed on 18 August 2022) and published literature [15,18,33]. 

To identify potential biomarkers for caries, we focused on the important metabolites defined by the following criteria: VIP > 1.0, fold change (FC) > 1.5 or < 0.6, and false discovery rate (FDR)-adjusted p-value from a Student’s t-test (FDR) < 0.05. In addition, the ROC analysis assessed diagnostic performances of the individual potential biomarkers. The area under the ROC curve (AUC) was calculated, and an AUC > 0.8 was considered a good diagnostic effect. A one-way ANOVA with Tukey’s post-hoc test was used to determine statistical differences in metabolites among the three groups (FDR < 0.05). For multiple biomarker panel, multivariate ROC analysis was performed. Multivariate ROC curve model building and performance evaluation were performed based on Monte Carlo cross-validation (MCCV) using balanced subsampling. For classification and feature ranking, the partial least squares discriminant analysis (PLS-DA) algorithm with two latent variables (LV) was applied. All analyses of metabolite concentration data were performed using the MetaboAnalyst software (version 5.0, www.metaboanalyst.ca, accessed on 12 August 2022). 

## 3. Results

### 3.1. Characteristics of Study Participants

Fifty-four participants with and without caries were recruited for this study (Table 1). Children in the control group had no decayed or filled teeth. The means of dft and dfs of the ECC group were 5.4 ± 2.7 and 14.0 ± 10.9, respectively. The Snyder test scores were higher in the ECC group than control group; however, the difference was not significant. This indicates that the participants in the ECC group were more susceptible to dental caries. Differences in sex, age, height, weight, drink and snack consumption frequency, and tooth brushing frequency were not significantly different between the groups.

### 3.2. Metabolic Profiling and Important Salivary Metabolites in Caries 

**Figure 1:**
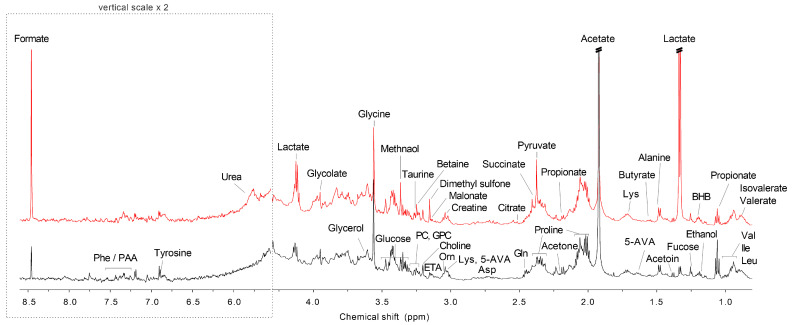
Representative ^1^H NMR spectra of saliva samples from study participants with caries (Red color) and the healthy control (Black color). Abbreviations: 3-HIA: 3-Hydroxyisovalerate; 5-AVA: 5-Aminopentanoate; Asp: Aspartate; BHB: 3-Hydroxybutyrate; ETA: Ethanolamine; Gln: Glutamine; GPC: Glycerophosphocholine; Ile: Isoleucine; Leu: Leucine; Lys: Lysine; Orn: Ornithine; PAA: Phenylacetate; PC: Phosphocholine; Phe: Phenylalanine; Val: Valine.

Figure 1 shows the representative ^1^H NMR spectra of saliva samples from the control and ECC groups. Forty-four metabolites were identified and quantified in the NMR spectra (Table S1). 

**Figure 2:**
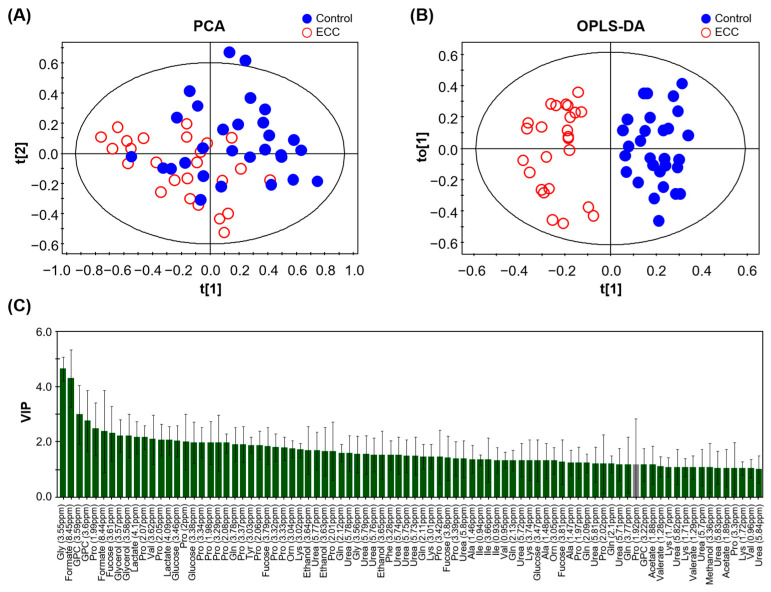
PCA (**A**) and OPLS-DA (**B**) score plots of ^1^H NMR spectra of saliva samples from study participants with caries (ECC) and the healthy control (Control). (**C**) VIP scores of the influential contributing variables in the OPLS-DA with the jack-knife standard error (VIP > 1.0). Green bars represent the values that satisfy the set criteria (VIP/VIP_cvSE_ >1.0), and gray bar denotes those that do not (VIP/VIP_cvSE_ < 1.0). Abbreviations: Ala: Alanine; Gln: Glutamine; Gly: Glycine; GPC: Glycerophosphocholine; Ile: Isoleucine; Lys: Lysine; Orn: Ornithine; Pro: Proline; Tyr: Tyrosine; Val: Valine.

PCA and OPLS-DA based on ^1^H NMR spectra were performed to confirm the metabolic differences between the control and ECC groups. The PCA revealed a marginal separation between the groups (Figure 2A). The goodness-of-fit of the PCA model was R^2^X = 0.487. 

OPLS-DA showed further improvement in discrimination between the two groups, with a goodness of fit R^2^Y = 0.87 and predictive quality Q^2^ = 0.485 (Figure 2B). This value was acceptable and reliable for a biological model (Q^2^ ≥ 0.4) [34]. In addition, the *p-*value of the CV-ANOVA was significantly lower than 0.05 (*p_CV-ANVOA_* = 0.0025), suggesting that the model was reasonable. Based on the VIP score obtained from the OPLS-DA model, we identified 21 metabolites that significantly contributed to the group separation (VIP >1.0, VIP/VIP_cvSE_ >1.0, Figure 2C). The levels of 17 metabolites in the saliva of participants with caries were significantly different between the control and ECC groups according to the prescribed criteria (FDR < 0.05, FC > 1.5, or FC < 0.6). Specifically, the levels of ethanol, formate, glycerophosphocholine (GPC), lactate, urea, and valerate were significantly higher in the ECC group than in the control group, whereas the levels of alanine, fucose, glutamine, glycine, isoleucine, lysine, ornithine, phenylalanine, proline, tyrosine, and valine were significantly lower. 

### 3.3. Multiple Biomarker Panel for Caries Diagnosis

**Figure 3:**
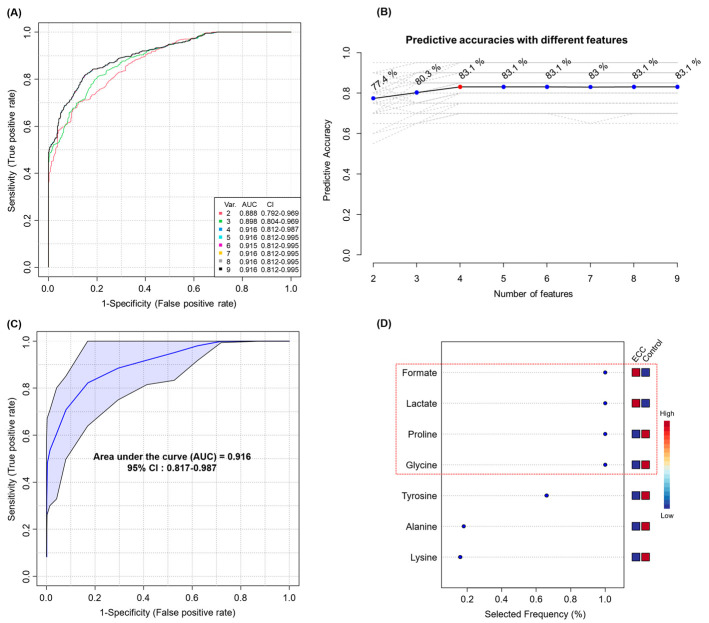
Multivariate ROC analysis. All ROC curves (**A**) and predictive accuracies (**B**) of five models created from nine biomarkers using different number of features. ROC curve (**C**) of model 3 generated using four features and the top significant features (**D**) predicted based on their frequencies of selection during cross-validation. Abbreviations: Var., variable; AUC, area under curve; CI, confidence interval.

ROC analysis revealed that the nine metabolites showed good diagnostic performance (AUC > 0.8) (Table 2 and Figure S1). The ROC curves for the 17 important metabolites are shown in Figure S1. Thereafter, we performed a multivariate ROC analysis with the nine selected potential biomarkers and created eight biomarker models considering different numbers of metabolites (2, 3, 4, 5, 6, 7, 8, and 9). Model 3, which was generated with four metabolites, had the highest AUC and prediction accuracy (AUC = 0.916, sensitivity = 85.7%, specificity = 80.8%, and predictive accuracy 83.1%), with the same performance as that of the model created using all nine biomarkers (Figure 3A–C). Figure 3D shows the top metabolites ranked by their mean importance of selection for model 4. The four biomarkers selected were formate, lactate, proline, and glycine. 

### 3.4. Levels of Selected Metabolites Based on the ICDAS Classification

Nine potential biomarkers were further analyzed based on ICDAS classification (Table 2). Patients in the ECC group were subdivided into ECC0 (not severe, ICDAS < 3) and ECC2 (severe, ICDAS ≥ 3). 

Formate and lactate levels were significantly increased in both the ECC0 and ECC2 groups compared to those in the control group. GPC levels significantly increased only in the ECC2 group compared to those in the control group. The levels of glycine, isoleucine, tyrosine, and lysine significantly decreased in both the ECC0 and ECC2 groups compared to those in the control group. Alanine and proline levels significantly decreased only in the ECC2 group compared to those in the control group. No metabolites were significantly different between the ECC0 and ECC2 groups.

### 3.5. Correlation Analysis 

The correlation between metabolite biomarkers and characteristics of the study participants was also investigated (Table 3). The characteristics included oral health characteristics (dfs, dft, and Synder test scores), drink and snack consumption frequency, and daily tooth brushing frequency. All the identified metabolites, except lactate, significantly correlated with both dfs and dft (*p* < 0.05). Lactate levels were significant but had relatively weak correlations with the Synder test scores (r = 0.305, *p* < 0.05). Formate and isoleucine levels were significant but had a weak correlation with the snack consumption frequency (r = 0.386, *p* < 0.05) and daily toothbrushing frequency (r = −0.320, *p* < 0.05), respectively

## 4. Discussion

This study aimed to determine the metabolic differences caused by dental caries and potential metabolite biomarkers in the saliva using ^1^H NMR-based metabolic profiling approach. Although several previous studies have reported that caries influences the salivary metabolome more strongly than sex or the dentition stage [21,30], we matched sex and age for groups in this study to minimize the effects of uncontrolled variables such as hormonal characteristics.

The OPLS-DA score plot of ^1^H NMR spectra showed that the salivary metabolome of patients with caries could be discriminated from that of the controls. Discriminatory buckets with VIP scores of 1 or higher were identified, and these regions were assigned to metabolites. Among these metabolites, nine potential salivary biomarkers (three upregulated and six downregulated) that could diagnose caries were identified. The levels of these biomarkers were significantly increased or decreased in both the ECC subgroups according to caries severity compared to those in the controls; however, no significant differences were observed between ECC0 and ECC2. Based on the multivariate ROC analysis, the best biomarker panel for caries diagnosis was identified as a combination of four discriminatory metabolites—formate, lactate, proline, and glycine. In general, panels of fewer biomarkers are preferred to many biomarkers because they are more robust, cost-effective, and less prone to over-fitting [32]. The selected panel showed the same diagnostic performance as that of the panel composed of all biomarkers, and it was higher than that of a single metabolite

To the best of our knowledge, no previous studies have investigated the correlation between metabolites, the number of caries, and dietary habits that affect caries development. In the present study, the concentrations of salivary metabolite biomarkers were significantly correlated with both dft and dfs. However, we did not observe meaningful associations between metabolite biomarkers and the drink and snack consumption frequency or tooth brushing frequency. These characteristics also exhibited weak associations with dft and dfs. It was reported that these snacking habits and low tooth brushing frequency of participants are associated with experiencing caries [35,36]. The lack of significant results in this study may be due to the small study population and a lack of significant differences in these characteristics between the control and ECC groups.

Some of the identified biomarkers have been reported to show significant changes in the saliva with dental caries. The main process that leads to caries formation is carbohydrate fermentation by dental plaque bacteria, which produces strong organic acids, such as lactate, formate, and pyruvate. These destructive organic acids lower the pH and cause the demineralization of tooth enamel. In this study, lactate and formate levels were significantly upregulated in the ECC group compared to those in the control group (FDR < 0.05). Lactate is the major end-product of glycolysis in cariogenic bacteria, such as *Streptococcus mutans* [37]. Previous studies have reported lactate as a metabolite marker for caries activity in the saliva [30,38] or dental plaque [39]. Notably, lactate levels showed relatively weak correlations with both dft and dfs. Lactate is reported to be more strongly associated with oral bacteria than with caries count [40], and we observed a significant positive correlation between lactate concentration and Snyder test scores in this study. Formate is also an acidic end-product of carbohydrate metabolism by caries-associated bacteria. Previous studies recommended formate as a potential biomarker related to the acidity of saliva [41,42] and reported a significantly higher concentration of formate in saliva with caries than that in caries-free saliva collected from children aged 6 to 12 years [40]. Furthermore, the positive correlation between formate and snack consumption frequency observed in this study may be related to the fact that formate is produced by oral bacteria via pyruvate formate lyase under aerobic and excess glucose conditions [42].

In addition to organic acids, GPC levels are upregulated in caries. GPC is a metabolite of phosphatidylcholine, which is the major phospholipid in saliva [43]. Phosphatidylcholine is associated with mucus glycoprotein, and its levels are higher in the saliva and plaques of caries-susceptible subjects than in those of caries-resistant subjects [44,45]. As the formation of hydrophobic bonds between bacteria and oral tissue is stabilized by a lipid-rich environment, high levels of salivary lipids can provide an oral environment for the facile adherence of bacteria [46,47]. However, limited information is available on the functions of phospholipids in saliva, and GPC has been reported to be positively associated with oral cancer, periodontal disease [48], dental prostheses, and missing teeth [49].

A noticeable observation of this result was that most amino acids were downregulated in the ECC group (FDR < 0.05). All downregulated potential biomarkers, including alanine, glycine, isoleucine, lysine, proline, and tyrosine, were amino acids. Amino acid levels in saliva were found to be relatively stable after food intake compared to those in the plasma and urine [50] and were not affected by circadian rhythms [51]. The presence of amino acids in saliva can enhance its ability to resist stress by buffering the changes in pH or osmolality [52]. Amino acids can be metabolized by supragingival saccharolytic bacteria into ammonia and amines, which contribute to acid neutralization during the development of dental caries [53,54]. In addition, some amino acids have been found to have cariostatic effects. Previous studies have reported increased glycine levels in the saliva of children without caries [55,56]. Glycine is a neutral amino acid and a major component of tooth collagen [57]. Dietary supplementation with glycine causes a reduction in caries development and the lipid content of teeth in rats [58] and humans [56]. The cariostatic effect of lysine, a dibasic amino acid, has been reported to be exerted systemically [59,60], and some studies have suggested that the lysine levels of caries-free subjects are higher than the lysine levels of those who have experienced caries in both whole saliva [55] and parotid saliva [61]. Therefore, we suggest that low levels of amino acids in saliva could indicate susceptibility to ECC. However, previous NMR-based metabolomics studies mainly focused on changes in organic acids and carbohydrates in saliva, and significant differences in amino acid composition have rarely been reported [21,30,31,62]. Contrary to the present results, one study reported an increase in amino acids in saliva affected by caries, although it was not statistically significant after Bonferroni correction [21]. This opposite result may be due to the fact that salivary amino acid levels are affected by age [63]. The aforementioned study was conducted with a relatively wide age range of study participants from 4 to 16 years. In a recent study involving children of a similar age range as in our study, low levels of amino acids, including proline, were found in the saliva of children with caries, which supports our present findings [62].

## 5. Conclusions

The present study investigated saliva samples from children with ECC using ^1^H NMR metabolomics, provided evidence of distinct metabolic differences, and identified potential metabolite biomarkers. These metabolites were significantly different between the control and ECC subgroups according to severity and were associated with caries experience. A metabolite biomarker panel composed of formate, lactate, proline, and glycine was established for ECC diagnosis, and it showed better diagnostic performance than a single metabolite. Overall, these findings emphasize the potential of salivary metabolites as diagnostic biomarkers of caries. Further studies should be conducted to validate these findings in a larger cohort and elucidate the associations between metabolite biomarkers and dietary factors that contribute to caries.

## Figures and Tables

**Table 1:** Clinical information of the study participants.

Characteristic		Control	ECC
Subjects (Male/Female)		29 (13/16)	25 (14/11)
Age (mean ± SD)		4.1 ± 0.8	4.4 ± 0.7
Body height (mean ± SD)		106.3 ± 7.1	106.4 ± 6.4
Body weight (mean ± SD)		17.8 ± 2.4	17.3 ± 2.4
dfs (decayed and filled teeth surfaces) (mean ± SD)		0	14.0 ± 10.9
dft (decayed and filled teeth) (mean ± SD)		0	5.4 ± 2.7
Snyder test score, n (%)	score 1	12 (41.4%)	8 (32.0%)
	score 2	10 (34.5%)	6 (24.0%)
	score 3	6 (20.7%)	7 (28.0%)
	score 4	1 (3.4%)	4 (16.0%)
ICDAS classification	Not severe (ICDAS score ≤3)		6 (24%)
	Severe (ICDAS score ≥3)		19 (76%)
Drink consumption frequency, n (%)	Rarely or Never	9	3
	1–2 times/week	10	12
	3–4 times/week	7	6
	5–6 times/week	3	3
	Daily	0	1
Snack consumption frequency, n (%)	Rarely or Never	0	0
	1–2 times/week	0	0
	3–4 times/week	11 (38.0%)	5 (20.0%)
	5–6 times/week	5 (17.2%)	3 (12.0%)
	Daily	13 (44.8%)	17 (68.0%)
Daily toothbrushing frequency, n (%)	1 time	1 (3.4%)	0
	2 times	11 (38.0%)	10 (40.0%)
	3 times	15 (51.7%)	15 (60.0%)
	4 times	2 (6.9%)	0

**Table 2:** Potential metabolite biomarkers for differentiating the samples from study participants with caries and a healthy control.

Metabolites	Fold Change	log_2_(FC)	t-test (FDR)	AUC	One-Way ANOVA	
					FDR	Tukey’s HSD
Glycine	0.33	−1.62	<0.001	0.903	<0.001	ECC0-Control; ECC2-Control
Formate	3.28	1.71	<0.001	0.883	<0.001	ECC0-Control; ECC2-Control
Tyrosine	0.52	−0.94	<0.001	0.843	<0.001	ECC0-Control; ECC2-Control
GPC	1.53	0.61	<0.001	0.839	<0.001	ECC2-Control
Lysine	0.52	−0.93	<0.001	0.839	<0.001	ECC0-Control; ECC2-Control
Isoleucine	0.47	−1.08	<0.001	0.836	<0.001	ECC0-Control; ECC2-Control
Proline	0.25	−1.98	<0.001	0.822	0.0015	ECC2-Control
Lactate	2.53	1.34	0.0067	0.817	0.0119	ECC0-Control; ECC2-Control
Alanine	0.60	−0.74	0.0017	0.803	0.0021	ECC2-Control

**Table 3:** Correlation analysis between nine potential metabolite biomarkers and clinical parameters. Pearson’s correlation coefficient (r) and the corresponding *p* values.

	dfs	dit	Snyder	Snack	Drink	Toothbrushing	Alanine	Formate	Glycine	GPC	Isoleucine	Lactate	Lysine	Proline	Tyrosine
dfs	1														
dft	0.906 (<0.001)	1													
Snyder	0.0026 (0.985)	0.139 (0.318)	1												
Snack	0.232 (0.092)	0.264 (0.053)	−0.122 (0.379)	1											
Drink	0.389 (0.004)	0.337 (0.012)	−0.092 (0.507)	0.123 (0.375)	1										
Toothbrushing	−0.042 (0.763)	0.0243 (0.862)	−0.217 (0.116)	0.228 (0.0971)	−0.119 (0.390)	1									
Alanine	−0.412 (0.0019)	−0.450 (<0.001)	0.150 (0.280)	−0.088 (0.525)	−0.115 (0.407)	−0.112 (0.418)	1								
Formate	0.487 (<0.001)	0.529 (<0.001)	−0.090 (0.521)	0.386 (0.004)	0.124 (0.371)	0.146 (0.294)	−0.415 (0.0018)	1							
Glycine	−0.468 (<0.001)	−0.555 (<0.001)	−0.038 (0.787)	−0.169 (0.222)	−0.100 (0.471)	−0.126 (0.364)	0.568 (<0.001)	−0.641 (<0.001)	1						
GPC	0.550 (<0.001)	0.605 (<0.001)	−0.017 (0.902)	0.153 (0.268)	0.078 (0.575)	0.206 (0.136)	−0.522 (<0.001)	0.487 (<0.001)	−0.661 (<0.001)	1					
Isoleucine	−0.414 (0.0018)	−0.522 (<0.001)	0.012 (0.929)	−0.262 (0.0557)	−0.161 (0.245)	−0.320 (0.0182)	0.562 (<0.001)	−0.534 (<0.001)	0.698 (<0.001)	−0.643 (<0.001)	1				
Lactate	0.116 (0.403)	0.198 (0.152)	0.305 (0.025)	−0.158 (0.253)	−0.047 (0.734)	−0.103 (0.459)	0.126 (0.365)	0.159 (0.249)	−0.264 (0.0535)	0.0779 (0.576)	−0.223 (0.105)	1			
Lysine	−0.403 (0.0025)	−0.461 (<0.001)	−0.071 (0.608)	−0.246 (0.0724)	−0.116 (0.402)	−0.144 (0.299)	0.472 (<0.001)	−0.648 (<0.001)	0.790 (<0.001)	−0.487 (<0.001)	0.595 (<0.001)	−0.289 (0.0341)	1		
Proline	−0.356 (0.0082)	−0.428 (0.001)	−0.083 (0.552)	−0.135 (0.330)	−0.060 (0.667)	−0.0442 (0.751)	0.508 (<0.001)	−0.545 (<0.001)	0.939 (<0.001)	−0.574 (<0.001)	0.546 (<0.001)	−0.235 (0.0879)	0.745 (<0.001)	1	
Tyrosine	−0.391 (0.0035)	−0.492 (<0.001)	−0.088 (0.525)	−0.263 (0.0542)	−0.217 (0.116)	−0.254 (0.0644)	0.360 (0.0076)	−0.643 (<0.001)	0.756 (<0.001)	−0.414 (0.0019)	0.752 (<0.001)	−0.365 (0.0067)	0.722 (<0.001)	0.608 (<0.001)	1

## Data Availability

Data are contained within the article or supplementary.

## Supplementary Materials

The following supporting information can be downloaded at: https://www.mdpi.com/article/10.3390/metabo13030356/s1, Figure S1: ROC curves for 17 important metabolites identified via multivariate and univariate statistical analyses.; Table S1: Identified and quantified salivary metabolites from ^1^H-NMR spectra.
