# Supplementary material for: Identification of a Biomarker Panel for Diagnosis of Early Childhood Caries Using Salivary Metabolic Profile
Source: Metabolites. 2023 Feb 27;13(3):356. doi: 10.3390/metabo13030356 (PMC10052657; doi:10.3390/metabo13030356)

Article

Identification of a Biomarker Panel for Diagnosis of Early Childhood Caries Using Salivary Metabolic Profile

Seonghye Kim [1,†], Yuri Song [2,3,†], Seyeon Kim [4], Siyeong Kim [3,5], Heesam Na [2,3,5], Sujin Lee [1], Jin Chung [2,3,5,*,‡] and Suhkmann Kim [1,*,‡]

Supplementary Data

Snyder's caries activity test Approximately 0.2 µL of the saliva was pipetted into melted Snyder's medium at 50 °C. After the addition of saliva to the medium, the tube was rotated for proper mixing of saliva and the medium and incubated at 37 °C for up to 72 h. Color changes were observed every 24 h. The rate of color change of Snyder's medium from green to yellow is indicative of caries activity. Snyder's test results were evaluated as follows: Score 1: no color change up to 72 h (negative caries activity); score 2: color change at 48–72 h (slight caries activity); score 3: color change at 24-48 h (moderate caries activity); and score 4: color change within 24 h (severe caries activity).

Table S1. Identified and quantified salivary metabolites from ^{1}H-NMR spectra.

Metabolites	Control (n=29)		ECC (n=25)					
					ECC0 (n=6)		ECC2 (n=19)	
	Mean (mM)	SD	Mean (mM)	SD	Mean (mM)	SD	Mean (mM)	SD
**3-Hydroxybutyrate**	0.0037	0.0014	0.0043	0.0016	0.0038	0.0012	0.0045	0.0017
**3-Hydroxyisovalerate**	0.0052	0.0017	0.0073	0.0025	0.0064	0.0037	0.0076	0.002
**5-Aminopentanoate**	0.0135	0.0044	0.0122	0.0041	0.0133	0.0023	0.0118	0.0046
**Acetate**	0.8554	0.2712	0.6242	0.1463	0.6216	0.1736	0.625	0.1419
**Acetoin**	0.0063	0.0043	0.0049	0.0042	0.0073	0.0077	0.0041	0.0021
**Acetone**	0.0034	0.0018	0.0041	0.0019	0.0037	0.0014	0.0043	0.002
**Alanine**	0.0157	0.0077	0.0094	0.0045	0.0124	0.0055	0.0085	0.0039
**Aspartate**	0.0167	0.0047	0.0121	0.0034	0.0127	0.0042	0.0119	0.0032
**Betaine**	0.002	0.0011	0.002	0.0011	0.0022	0.001	0.0019	0.0012
**Butyrate**	0.0116	0.0048	0.0151	0.0053	0.0122	0.0041	0.016	0.0054
**Choline**	0.0021	0.0013	0.0019	0.0008	0.0026	0.0009	0.0017	0.0007
**Citrate**	0.0048	0.0018	0.0045	0.0017	0.0051	0.0027	0.0043	0.0014
**Creatine**	0.006	0.0013	0.0058	0.0014	0.0063	0.0009	0.0056	0.0015
**Dimethyl sulfone**	0.0021	0.0011	0.0035	0.0019	0.0029	0.0015	0.0037	0.002
**Ethanol**	0.012	0.0085	0.0201	0.0095	0.0153	0.009	0.0217	0.0094
**Ethanolamine**	0.0116	0.0081	0.0136	0.0053	0.0113	0.0055	0.0144	0.0051
**Formate**	0.2164	0.2457	0.7101	0.3461	0.6104	0.4358	0.7416	0.3203
**Fucose**	0.0164	0.0079	0.0101	0.0029	0.0101	0.0034	0.0101	0.0029
**Glucose**	0.0272	0.0091	0.0406	0.0112	0.0409	0.0087	0.0406	0.0121
**Glutamine**	0.0356	0.0253	0.0158	0.0083	0.0216	0.0094	0.014	0.0072
**Glycerol**	0.0238	0.0185	0.0321	0.0095	0.0325	0.0112	0.032	0.0092
**Glycerophosphocholine**	0.0024	0.001	0.0037	0.0009	0.003	0.0009	0.0039	0.0008
**Glycine**	0.1152	0.0636	0.0375	0.025	0.0619	0.0387	0.0298	0.0126
**Glycolate**	0.0273	0.0117	0.0373	0.0116	0.0368	0.0148	0.0374	0.0109
**Isoleucine**	0.0044	0.0021	0.0021	0.0009	0.0025	0.0013	0.002	0.0008
**Isovalerate**	0.0019	0.0006	0.002	0.0007	0.0019	0.0007	0.002	0.0007
**Lactate**	0.0382	0.0416	0.0967	0.0939	0.116	0.1367	0.0906	0.0801
**Leucine**	0.0056	0.0021	0.0038	0.0013	0.0045	0.0011	0.0035	0.0013
**Lysine**	0.0187	0.0087	0.0098	0.004	0.0115	0.0048	0.0093	0.0037
**Malonate**	0.0048	0.0018	0.0042	0.0019	0.0045	0.0023	0.0041	0.0018
**Methanol**	0.0265	0.0133	0.0419	0.0183	0.0282	0.0091	0.0462	0.0185
**Ornithine**	0.0104	0.006	0.0053	0.0032	0.0072	0.0039	0.0048	0.0029
**Phenylacetate**	0.0038	0.0018	0.004	0.0018	0.0032	0.0015	0.0043	0.0018
**Phenylalanine**	0.009	0.0035	0.0059	0.0019	0.0055	0.002	0.006	0.0018
**Phosphocholine**	0.0027	0.0013	0.0034	0.0016	0.0024	0.0012	0.0037	0.0016
**Proline**	0.1275	0.1135	0.0323	0.0273	0.0539	0.0497	0.0254	0.0101
**Propionate**	0.0642	0.0304	0.0624	0.02	0.0595	0.0246	0.0633	0.019
**Pyruvate**	0.0133	0.0113	0.0201	0.0154	0.0272	0.0225	0.0179	0.0124
**Succinate**	0.0069	0.0052	0.0106	0.0049	0.0089	0.0031	0.0111	0.0053
**Taurine**	0.0227	0.0065	0.0247	0.0076	0.0224	0.0071	0.0254	0.0078
**Tyrosine**	0.0205	0.0092	0.0107	0.0041	0.0121	0.0038	0.0102	0.0041
**Urea**	0.1919	0.1732	0.4093	0.2733	0.2749	0.2271	0.4517	0.2781
**Valerate**	0.0076	0.0047	0.0121	0.0063	0.0102	0.0037	0.0127	0.0068
**Valine**	0.0042	0.0024	0.0022	0.001	0.0026	0.0012	0.0021	0.0009

Figure S1. ROC curves for 17 important metabolites identified via multivariate and univariate statistical analyses.

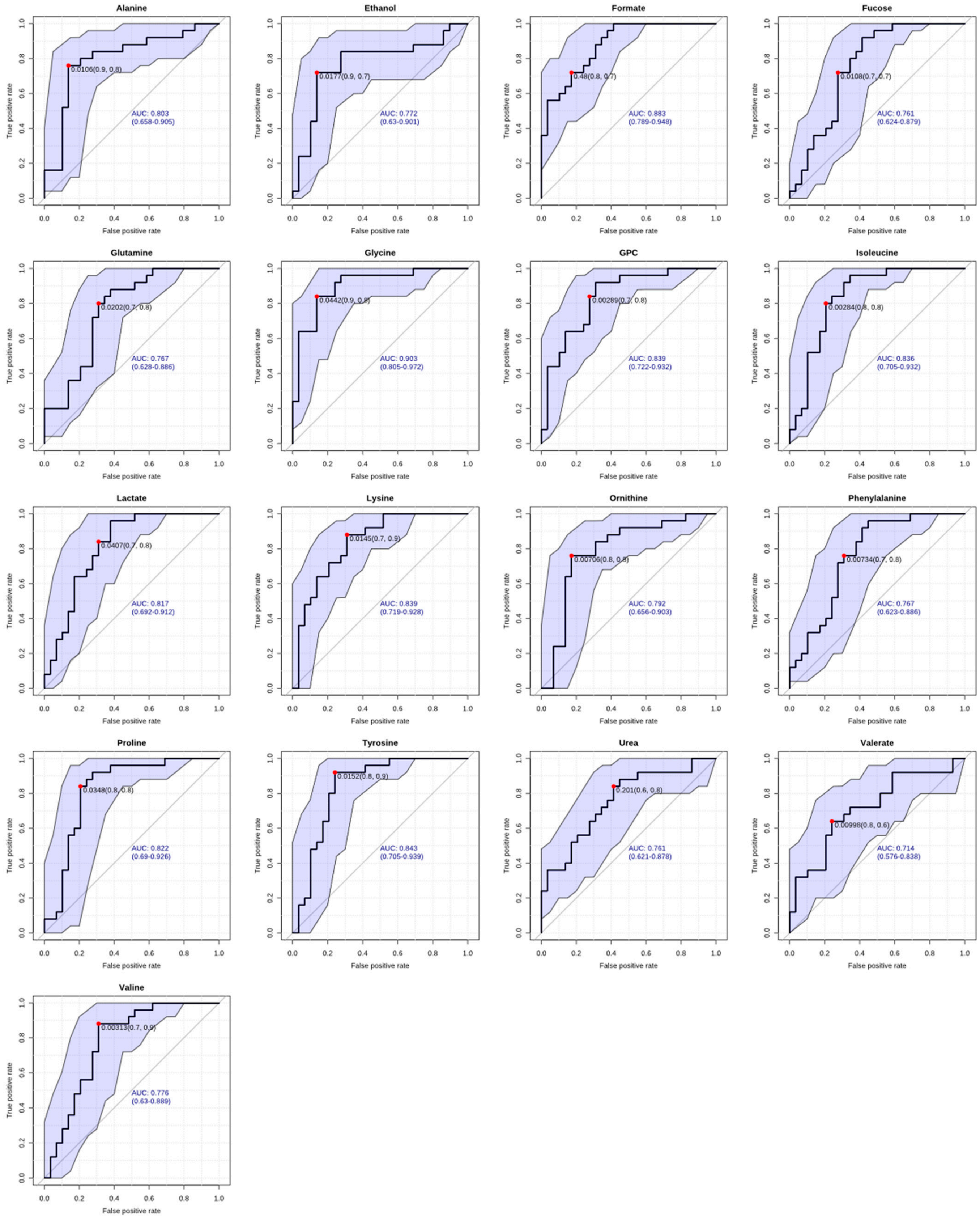

Supplement: Supplementary file 1 [file metabolites-13-00356-s001.zip › metabolites-2236592-supplementary.pdf]
